# Stroke-like Episodes in Inherited Neurometabolic Disorders

**DOI:** 10.3390/metabo12100929

**Published:** 2022-09-30

**Authors:** Natalia Będkowska, Aneta Zontek, Justyna Paprocka

**Affiliations:** 1 Students’ Scientific Society, Department of Pediatric Neurology, Faculty of Medical Sciences in Katowice, Medical University of Silesia, 40-752 Katowice, Poland; 2 Department of Pediatric Neurology, Faculty of Medical Sciences in Katowice, Medical University of Silesia, 40-752 Katowice, Poland

**Keywords:** stroke-like episode, stroke-like lesion, inherited errors of metabolism, neurometabolic disorders, MELAS, MERRF, Leigh syndrome, Kearns–Sayre syndrome, urea cycle disorders, congenital disorders of glycosylation, organic acidemia, organic aciduria, MRI, EEG, histopathology

## Abstract

Stroke-like episodes (SLEs) are significant clinical manifestations of metabolic disorders affecting the central nervous system. Morphological equivalents presented in neuroimaging procedures are described as stroke-like lesions (SLLs). It is crucial to distinguish SLEs from cerebral infarction or intracerebral hemorrhage, mainly due to the variety in management. Another significant issue to underline is the meaning of the main pathogenetic hypotheses in the development of SLEs. The diagnostic process is based on the patient’s medical history, physical and neurological examination, neuroimaging techniques and laboratory and genetic testing. Implementation of treatment is generally symptomatic and includes L-arginine supplementation and adequate antiepileptic management. The main aim of the current review was to summarize the basic and actual knowledge about the occurrence of SLEs in various inherited neurometabolic disorders, discuss the possible pathomechanism of their development, underline the role of neuroimaging in the detection of SLLs and identification of the electroencephalographic patterns as well as histological abnormalities in inherited disorders of metabolism.

## 1. Introduction

Stroke-like episodes (SLEs) are one of the most basic clinical manifestations of neurometabolic disorders, especially mitochondrial myopathy, encephalopathy, lactic acidosis and stroke-like episodes syndrome (MELAS); however, they could occur in other inherited disorders of metabolism such as Leigh syndrome (LS), Kearns–Sayre syndrome (KS), Myoclonus epilepsy with ragged red fibers syndrome (MERRF), urea cycle disorders, organic acidemias, lysosomal storage diseases, congenital disorders of glycosylation (CDG) as well [1,2,3,4]. It is crucial to distinguish SLE from ischemic stroke due to different etiopathogenesis and treatment. Imaging techniques could reveal changes called stroke-like lesions that are the morphological equivalent of SLEs and are located especially in the posterior brain areas [1,5,6,7,8].

SLEs are a cardinal phenotypic feature of various mitochondrial disorders and may occur already in early childhood, but the average onset is adolescence or early adulthood, about 40 years old [1,2,3,4,5,6,9]. Clinically, SLEs mimic ischemic stroke or intracerebral bleeding. SLEs are frequently associated with other abnormalities, such as epileptic seizures, ataxia, migraine headaches, impaired hearing, visual impairment, amnesia, cognitive impairment, psychosis, hallucinations, confusional state or coma. Clinical manifestations of SLE include confusion; hyperthermia; and focal neurological disabilities such as hemiparesis, hemiplegia, dysphagia or hemianopsia [1,4,10,11,12,13,14,15,16].

Pathogenesis of SLEs is still unexplained, but it was confirmed that the cerebral lesion equivalent to an SLE is vasogenic edema. In most cases, SLEs develop spontaneously, but single cased could be triggered by drugs, for example, zonisamide or phenytoin [1,4,5,6,10,12,17,18,19,20,21,22]. The pathomechanism of SLEs remains still unclear; however, there are some hypotheses that may propose an explanation for the most probable ways of SLL development [1,5,6]. In general, the SLE’s pathogenesis is associated with the dysfunction of mitochondria [1,5,17,23]. Morphological equivalents of SLEs are described as SLLs and could be detected via neuroimaging techniques, especially magnetic resonance imaging (MRI). SLLs are pathological changes observed mainly in the posterior brain regions, especially in the location of temporal lobes [7,8,24]. Generally, SLLs evolve in areas incompatible with the vascular territory, but in some cases, they can be connected with vasogenic edema due to increased vascular permeability spreading to the local cortical regions [1,5,7,8,17,18].

However, a few principal conceptions could be an explanation of SLEs pathogenesis, and the most probable point concerns the concomitance of current hypotheses. It seems to be crucial to understand the main issues to avoid inappropriate treatment methods [16,22,25]. Signs and symptoms associated with the occurrence of SLEs are, in general, connected with the lesion site. They are also characterized by reversibility and a tendency to recur. These changes lead to progressive complications in brain functioning and may also result in neuronal atrophy [1,5,7,8]. Neuronal dysfunction in metabolic strokes seems to be connected with various alterations such as endothelial dysfunction, a tendency to hypercoagulation, cerebral perfusion dysfunction due to aggregation of metabolites and secondary neurotoxicity without rupture or obstruction of larger brain vessels [1,5,6,13,16,17,18,19,20,21,26,27].

The main purpose of the current review was to assume and discuss the principal hypotheses connected with SLEs pathogenesis. We also considered the main neurometabolic disorders associated with SLEs occurrence, specific neuroimaging changes and the electroencephalographic (EEG) patterns coexisting with various metabolic errors of metabolism. Performing a continuous EEG could especially improve the accuracy of diagnostic management in patients with inborn errors of metabolisms and reveal epileptic discharges secondary to increased specific metabolites due to the epileptogenic hypothesis [1,4,5,6,10,12,17,23]. It seems to be crucial to differentiate SLEs mainly from ischemic stroke due to distinct necessary treatment procedures. Due to the etiology of SLEs in the pediatric population, it seems to be crucial to concern various metabolic disorders that have an impact on the functioning of the central nervous system (CNS).

## 2. Materials and Methods

The current review paper was created by incorporating appropriate articles after searching for references using presented search terms in various configurations: “neurometabolic disorder”, “stroke-like episode”, “stroke-like lesion”, “stroke mimic”, “childhood”, “children”, “pediatric population”, “inborn errors of metabolism”, “MELAS”, “MERRF”, “Kearns Sayre syndrome”, “Leigh syndrome”, “Dihydropteridine reductase deficiency”, “DHPR”, “Succinic semialdehyde dehydrogenase deficiency”, “SSADH”, “urea cycle disorders”, “Carbamoyl phosphate synthetase 1 deficiency”, “CPS1”, “Ornithine transcarbamylase deficiency”, “OTC”, “Citrullinemia”, “Organic aciduria”, “Organic acidemia”, “methyl malonic aciduria”, “propionic aciduria”, “isovaleric aciduria”, “Fabry’s disease”, “Cystinosis”, “Thiamine-responsive megaloblastic anemia”, “congenital disorder of glycosylation”, “PMM2 CDG”, “MRI”, “neuroimaging”, “EEG”, “electroencephalogram”, “pathology”, “histopathology”, “prevalence” and “frequency of SLE”. Databases that were used during the investigation include PMC, NCBI, Medline, Scopus and Google Scholar. Articles published in English, German, French, Spanish or Polish were taken into consideration, and information from abstracts of manuscripts in other languages or papers without full-text access could be cited. All article types were taken into account: clinical trial, meta analysis, case report, case series, review, systematic review, books and documents. The aforementioned disorders were ranged based on the occurrence and prevalence in infant generation and also the frequency of stroke-like episodes. The current knowledge from available articles was used to estimate the probability of occurrence of SLEs. Based on MRI and EEG examination, differences between ischemic stroke and stroke-like episodes were underlined.

## 3. Results

### 3.1. Neurometabolic Mitochondrial Disorders Associated with SLEs

#### 3.1.1. MELAS Syndrome

MELAS is a mitochondrial neurometabolic disorder that could affect other systems, such as the cardiovascular system. In general, its inheritance is associated with *MTTL1 (#590050)* gene mutation, although some others connected with nuclear genes mutation are observed [2,5,9,13,14,15,16,27,28]. Pathogenesis of this disorder is related to the accumulation of deteriorative mitochondria that result in decreasing energy production, nitric oxide (NO) deficiency, angiopathy and dysfunction of the endothelium [5,9,14,15,16,17,23,27]. MELAS is characterized by broad clinical manifestations, including SLEs, epileptic seizures, height deficiency, dementia, episodes of headache, metabolic acidemia due to increased lactate level, diabetes mellitus, hearing loss and muscle weakness [14,15,16]. Symptomatic treatment is generally implemented. Methods such as supplementation of L-arginine, carnitine or Coenzyme Q10 were proposed. The results suggest that L-arginine implementation can reduce the risk of SLEs occurrence or the severity of presented episodes [25,29,30,31,32]. According to the retrospective study by Yi Shiau Ng, the most pathogenic mutation variant connected with the occurrence of SLE is the m.3243A > G variant in *MT-TL1.* This mutation concerns about 80% of patients with MELAS [6]. The prevalence of the m.3243A  >  G mutation is estimated in 3,5/100 000 cases [6]. There are three principal explanations of SLL pathogenesis in MELAS. Firstly, the vascular hypothesis seems to be associated with mitochondrial angiopathy caused by the proliferation of mitochondria in the smooth muscle layers of the brain arterioles [13,17,18,19,20,21]. For the epileptogenic hypothesis, there is no doubt that metabolic disorders are connected with a higher risk of seizure development. The higher potentiality to evolve in an inappropriate neuronal activity may be associated with brain cell dysfunction due to neuronal hyperexcitability [5,12,23,26,27,33]. Prolonged epileptic activity may potentially result in spreading vasogenic edema to the enclosing cortical areas and cause brain damage [5,16,23]. For the metabolic hypothesis, the generalized cytopathic hypothesis, also described as mitochondrial cytopathy, is another issue that may explain the possibility of SLEs occurrence and development. In general, it is associated with astrocyte damage due to mitochondrial dysfunction resulting in a lack of energy and lactic acidemia [1,5,6,17]. SLLs in MELAS have a tendency to repeat and reverse and are characterized by slow progress. Prevention of SLEs in MELAS may depend on the implementation of amino acids such as taurine or L-arginine [1,5,6,8,16,30,31]. Neurological impairments are present in more than 80% of individuals with MELAS, and the frequency of SLEs in this condition is about 60%−80% [5,16,28].

#### 3.1.2. MERRF Syndrome

The major genetic causes of mitochondrial epilepsy are mitochondrial DNA mutations. One of these diseases is MERRF—a rare mitochondrial multisystem disorder. Often the first symptom is myoclonus. Additionally, we observed generalized epilepsy, ataxia, weakness, exercise intolerance and dementia [34,35,36,37]. Onset can occur from childhood and adulthood. Common signs are ptosis, hearing loss, optic atrophy, cardiomyopathy, Wolff–Parkinson–White (WPW) syndrome and peripheral neuropathy. Pigmentary retinopathy and optic nerve neuropathy can also occur [34,35,36,37]. Most cases have the common mutation *m8344A > G* in the *MTTK* gene, but MERRF has also been linked to other mtDNA point mutations [34]. Radiographic features of MERRF are cerebral atrophy, cerebellar atrophy, high T2 white matter signal, globus pallidus atrophy and dentate nucleus calcification [38].

#### 3.1.3. Kearns–Sayre Syndrome

KSS is a neurodegenerative disorder, mostly diagnosed before the age of 20. Symptoms of this disease are pigmentary retinopathy, which leads to vision loss, ophthalmoplegia and ptosis. Moreover, we observed cardiac conduction defects, cerebellar dysfunction, or elevated cerebrospinal fluid (CSF) protein. This multisystem disorder is caused by large heteroplasmic deletions of the mtDNA. Leukoencephalopathy in KSS was also demonstrated by MRI to show cerebral, cerebellar and brainstem atrophy. T2 signals include subcortical prolongation with subcortical calcifications, with or without bilateral basal ganglia calcific deposits [39,40,41,42].

#### 3.1.4. Leigh Syndrome

LS, also described as subacute necrotizing encephalomyelopathy, is a genetic metabolic disorder that affects especially the functioning of the CNS. The prevalence of this condition is not high. In general, LS affects children at the age of 2 years or younger. Its inheritance is autosomal recessive and caused by heterogenous mitochondrial mutations that could be connected with various components of mitochondrial respiratory chain complexes and genes that encode mitochondrial tRNA or those associated with pyruvate dehydrogenase complex mutations [43,44]. In general clinical symptoms include neurological manifestations such as hypotonia, ataxia, dystonia, nystagmus, strabismus, ophthalmoplegia, retinopathy, ptosis, epileptic seizures, retardation of development, dysfunction of the cardiorespiratory system dysfunction, and gastrointestinal and hepatic impairments [43,44]. Laboratory tests in patients with LS demonstrate an increase in lactate blood and/or CSF levels [43,44]. Morphogenetic changes are associated with bilateral focal necrotic lesions mainly localized in the area of the thalamus, the brainstem and posterior parts of the spinal cord [45]. Morphological equivalents of SLEs in LS located in basal ganglia/thalamus are characterized by hyperintensity on T2-weighted and fluid-attenuated inversion recovery (FLAIR) sequences, isodensity on apparent diffusion coefficient (ADC)/ diffusion-weighted imaging (DWI) due to vasogenic edema [45].

### 3.2. Other Inborn Errors of Metabolism

SLEs could mimic stroke and should always be differentiated from typical stroke because it requires specific treatment. Patients with any type of mitochondrial disease may virtually present with SLEs [2,3,10,11].

#### 3.2.1. Dihydropteridine Reductase (DHPR) Deficiency

DHPR is one of the enzymes catalyzing the biosynthesis and regeneration of tetrahydrobiopterin (BH4), an essential cofactor that is necessary for the synthesis of dopamine, serotonin, norepinephrine and epinephrine. Some patients experienced stroke despite optimal treatment and metabolic control. The reason for this is unknown, but stroke could be associated with a very low level of 5-Methyltetrahydrofolate (5-MTHF) in CSF [46].

#### 3.2.2. Succinic Semialdehyde Dehydrogenase (SSADH) Deficiency

SSADH deficiency is an autosomal recessive disorder of gamma-aminobutyric acid metabolism. The 4-hydroxybutyric acid, which is accumulated, is neurotoxic; the oxidative stress processes and dysfunction of Gamma-aminobutyric acid (GABA) neuro-transmission are also impacted, but the exact pathophysiology of SLEs in patients with SSADH is unclear [47]. The other symptoms of this disease are mental retardation, autism, ataxia and epileptic seizures. The metabolic stroke must be suspected in children with developmental delay presenting focal neurological deficits and who have also had seizures. These children need to be covered by urine organic acid screening to exclude this disorder. There is no curative therapy [47,48].

### 3.3. Urea Cycle Disorders (UCDs)

UCDs are rare inborn errors that can present with a multitude of different symptoms and signs. Dysfunction of the urea cycle could involve stroke and SLEs. SLEs may be observed in carbamoyl phosphate synthetase I (CPS1) deficiency, ornithine transcarbamylase (OTC) deficiency and citrullinemia [3,49,50,51,52,53,54,55,56,57,58,59,60,61,62,63,64,65,66]. The pathogenesis of stroke-like events in patients affected by urea cycle disorders is unclear. The accumulation of ammonium impairs amino acid metabolism, and neurotransmission dysfunction oxidative stress could be responsible for neurotoxicity [49,50]. During the developmental period, the brain is more susceptible to damage due to hyperammonemia that leads to neurotransmitter pathways alternation, dysfunction in amino acids metabolism, increased synthesis of NO, production of reactive oxygen species (ROS), brain cells death and dysfunction in molecular transduction processes [49,50]. These changes result in various neurological impairments such as cerebral atrophy, secondary enlargement of brain ventricles, damage to the myelin and neuronal cell growth retardation [49,50,51]. Hyperammonemia results in brain cytotoxicity due to inhibition of gamma-ketoglutarate–dehydrogenase—one of the tricarboxylic cycle enzymes and activation of NMDA N-methyl-D-aspartate receptor. These alterations could explain the occurrence of SLLs in UCDs [49,50].

#### 3.3.1. Carbamoyl Phosphate Synthetase 1 (CPS1) deficiezncy

CPS1 deficiency is most common in the pediatric population and can also present in adulthood; however, it is rare. In the neonatal period, symptoms are nonspecific, for example, poor feeding, vomiting, somnolence and irritability. Those who present in adulthood usually have milder symptoms than children and also may be asymptomatic. MRI shows cortical injuries, including acute ischemia, ventricular dilatation and myelination defects [54,55,56,57].

#### 3.3.2. Ornithine Transcarbamylase Deficiency (OTC)

OTC deficiency is the most common UCD inherited as an X-linked disease. The classic presentation of OTC deficiency in hemizygous males in the first weeks after birth is a catastrophic illness. The OTC deficiency may present as hyperammonemia encephalopathy, seizures and rarely as SLEs. OTC deficiency should be considered in a child with recurrent SLEs, and encephalopathies with hyperammonemia, as early treatment prevents recurrence, morbidity and mortality [58,59,60,61].

#### 3.3.3. Citrullinemia

Pathomechanism of SLEs is not clear, but we suspect accumulation of toxic metabolites or disruption in mitochondrial function. It is a rare cause but has already been described as the same case of SLE, which is correlated with citrullinemia; these diagnoses are confirmed by MRI and blood tests. Urea cycle disorders should always be included in differential diagnoses of unexplained stroke during infancy and childhood. Citrullinemia type I is an autosomal recessive disorder associated with mutations of argininosuccinate synthase and secondary accumulation of ammonia [62,63,64,65,66].

The features of various neurometabolic disorders characterized by the occurrence of SLEs include genetic and molecular basis, clinical manifestations, MRI and EEG results, onset and prevalence and treatment methods (Table 1) [2,3,4,7,8,9,11,13,14,15,16,23,24,25,26,27,28,29,30,31,32,33,34,35,36,37,38,39,40,41,42,43,44,45,46,47,48,49,50,51,52,53,54,55,56,57,58,59,60,61,62,63,64,65,66].

### 3.4. Organic Acidurias

Typical forms of methylmalonic acidemia (MMA)/propionic acidemia (PA) include neonatal onset. Symptoms usually start as early as the second day of life without dehydration, weight loss, temperature instability, neurological involvement with muscular hypo- or hypertonia and, in the worst cases, coma and seizures. Laboratory findings are metabolic acidosis and ketosis, elevated anion gap and hyperammonemia. Late-onset cases of MMA and PA may present at any age [3,67,68]. Acute metabolic decompensation could be detected by performing plasma and CSF laboratory tests by defying increased fluid/plasma ratios for various metabolites such as lactate, alanine or glutamine and normal glycine ratio [68,69,70]. In the case of assembling the accuracy of the diagnostic process, MRI neuroimaging should be implemented (Table 2) [3,67,68,69,70,71,72,73].

### 3.5. Lysosomal Storage Disorders

#### 3.5.1. Fabry’s Disease

Fabry’s disease (FD) is an inherited metabolic disease that results from lack of the enzyme α-galactosidase due to *GLA* gene mutation. FD is inherited as an X-linked trait. FD is a lysosomal storage disease due to the accumulation of the sphingolipid globotriaosylceramide (GL-3 or Gb3) and its deacetylated derivative lyso-globotriaosylceramide (lyso-GL-3 or lyso-Gb3). The classic mechanism of stroke in FD includes endothelial dysfunction, higher production of ROS and a prothrombotic state [2,74,75]. FD is responsible for less than 1% of cryptogenic ischaemic strokes in young adults. There is a casual relationship between FD and ischaemic stroke, but the underlying mechanisms are not clear. Ischaemic stroke is the most frequent subtype, but intracerebral hemorrhages and cerebral venous thrombosis could also occur. The posterior circulation seems to be predominantly affected. The onset of the stroke is about 20–50 years. Neuroradiological features of MRI are dolichoectasia of the basilar artery and bilateral T1-weighted hyperintensity of the pulvinar [2,74,75,76,77]. The presented disease is connected to the *GLA* mutation. Pathogenesis of neurological events in patients with FD is connected to both small and large brain vessel angiopathy due to dysfunction of the endothelium, cerebral hyperperfusion, production of reactive oxygen species and increased tendency to thrombosis [74,75,76,77].

#### 3.5.2. Cystinosis

Cystinosis is a rare autosomal recessive disorder. The occurrence of mutation in the *CTNS* gene causes cystine accumulation in the cells, especially in lysosomes. Kidneys are affected most frequently, which results in renal Fanconi syndrome. The aforementioned condition also has an influence on the central nervous system. Cystinosis encephalopathy and SLEs could be observed. The frequency of these neurological complications is not high. Performing computed tomography (CT) imaging during SLEs could reveal calcifications located in the area of the hippocampus. Cortical or central atrophy is also observed but is not correlated with symptoms [78,79,80] (Table 3) [2,74,75,76,77,78,79,80].

### 3.6. Thiamine-Responsive Megaloblastic Anemia

Thiamine-responsive megaloblastic anemia (TRMA syndrome), also known as Rogers syndrome, is usually associated with non-autoimmune early-onset diabetes mellitus, anemia and sensorineural deafness due to autosomal recessive mutations in the *SLC19A2* gene that encodes thiamine transporter. Mutation in the *SLC19A2* gene is associated with loss-of-function in plasma membrane thiamine-transporter I. Vitamin B1 is transported into cells via two transporters: THTR1, associated with the *SLC19A2* gene, and THTR2, which is encoded by the *SLC19A3* gene. Loss-of-function mutation of *SCL19A2* is connected with dysfunction of pancreatic beta-islet cells, ear cells and stem cells of hematopoiesis due to high expression of THTR1 thiamine transporter on aforementioned units [81,82,83,84,85]. TRMA is commonly diagnosed during infancy or early childhood. Apart from typical symptoms, stroke or stroke-like episodes could also be observed, even with homozygous null mutations. Basic therapy is pharmacological doses of thiamine (25–75 mg per day) [81,82,86,87,88,89]. However pathogenesis of stroke-like episodes and other neurological features in patients with TRMA is still unclear; it may be connected with complex I of respiratory chain dysfunction due to thiamine deficiency, induction of oxidative stress and reduction in oxidative phosphorylation due to the lower synthesis of thiamine pyrophosphate, which is the cofactor of PDHE1α [81,82,88,90]. The prevalence of TRMA is unknown. This rare condition has been reported in 30 families around the world. Neurological manifestations are observed in 20–40 % of patients with TRMA in their early childhood [81,82,87,88]. THTR2 transporter seems to be more essential for thiamine brain active transport than the THTR1 channel [88,90] (Table 4) [81,82,83,84,85,86,87,88,89,90].

### 3.7. Congenital Disorders of Glycosylation—Phosphomannomutase 2 Deficiency

Among the various types of post-translational modification of proteins, glycosylation seems to be one of the most significant in human polypeptides. Glycoproteins play a key role in many organic processes, then defects in the synthesis of glycans or their adjunction to lipids or proteins result in a broad spectrum of clinical manifestations, including various neurological impairments including cerebellar syndrome (ataxia, dysarthria, nystagmus) [91,92]. The most common congenital disorder of glycosylation is phosphomannomutase 2 deficiency (PMM2-CDG), which is characterized by chronic cerebellar atrophy as the most frequent clinical manifestation. Other clinical features include migraine, epileptic seizures and SLEs [91,92,93,94]. Secondary to the atrophy of the cerebellum, patients develop ataxia; PMM2-CDG is associated with the *CACNA1A* gene gain-of-function mutation that encodes the voltage-gated CaV2.1 channel [93,94]. The presented mutation leads to hypoglycosylation of the α1A and α2α CaV2.1 channel subunits. A similar genetic alteration could also be observed in patients with ataxia and Familial Hemiplegic Migraine (FMH), the paroxysmal neurological disorder connected with mutations in ion transporters: CACNA1A that encodes the alpha 1A subunit of CaV2.1 voltage-gated channel [92,93,94,95]. Other mutations associated with FHM include ATP1A2, which encodes the alpha2 subunit of Na^+^, K^+^-ATPase pump or SCN1A (responsible for encoding the NaV1.1 channel alpha1 subunit). These molecular alterations could result in dysfunction of N-glycosylation that could lead to SLE. Symptoms of this rare disorder include severe headache and aura manifestations such as unilateral weakness, hemianopia, aphasia, paraesthesia or vertigo [92,94,95,96]. Ataxia in PMM2 CDG disorder is connected with the hypoglycosylation of the alpha-beta-δ subunit and leads to the opening of the CaV2.1 voltage-gated channel. According to studies, there are no significant genetic differences in patients with PMM2 CDG who are affected by SLE in comparison to ones who do not suffer from this clinical manifestation [93,94,97]. As a consequence of the unspecificity of SLE clinical manifestations, EEG results and radiological and laboratory test findings, there is a requirement to create specific investigation models to improve the whole diagnostic process. Due to various research, migraine, focal neurologic impairments, slower unilateral EEG patterns and hyperpyrexia with no indications of infectious disease revealed in laboratory tests may suggest the occurrence of SLE in patients with PMM2 CDG [92,94]. Correlation between head injury and occurrence of SLE seems to be associated with alternation of N-glycosylated mechanoreceptors functions due to impairment of the glycosylation process. Another propound cause of SLE in PMM2 CDG is viral infectious disease [94] (Table 5) [91,92,93,94,95,97].

### 3.8. The Role of Neuroimaging in Diagnosis of SLE

In addition to medical history, physical examination and laboratory tests, neuroimaging plays a key role in the diagnostic process of SLEs in inborn errors of metabolism. MRI is a specific technique to detect and monitor brain lesions in patients with various neurometabolic disorders. It also provides an accurate decision for further management. Typical SLLs in MELAS include lesions in the cerebral cortex and subcortical white matter. Thalamus may also be affected. Particularly cortical lesions are multiple and asymmetrical. MRI imaging seems to be the most specific and accurate way to distinguish SLEs from other neurological events. MRI findings in the acute stage of SLLs include cortical swelling presenting with hyperintensity on T2-weighted and T2 FLAIR sequences. In T1-weighted, after applying contrast, the patchy or linear enhancement could be observed in cortical lesions. The subacute stage is characterized by developing gyriform hyperintensity on T1-weighted sequence and hypointensity on T2-weighted/T2 FLAIR due to stratified cortical necrosis. The chronic stage encompasses cerebral encephalomalacia, gliosis and atrophy of the affected regions [1,7,8,24]. SLLs always present high signals on DWI. In ADC, signals alternately change or mix in different periods. After the acute phase, the ADC value can return to normal. These changes may be associated with the different levels of mitochondrial electron transport chain dysfunction. Moderate cellular impairment with vasogenic edema results from mild energy failure. Irreversible cellular dysfunction responsible for cytotoxic edema is caused by a severe decrease in mitochondrial energy production [1,7,8,24]. MELAS is characterized by an increased lactate peak in the lesion area and decreased N-acetylaspartate peak on proton magnetic resonance spectroscopy (1H-MRS). These changes are not specific and could also occur in stroke. A lactate peak on MRS shows anaerobic metabolism, but lactate signals could be detected in normal cerebrospinal fluid in about ⅓ of patients. MRS can be used to diagnose and monitor the course of MELAS [7,8,24]. Similar perfusion-weighted imaging (PWI) and arterial spin labeling (ASL) could demonstrate microscopic hemodynamic information of the brain and evaluate cerebral perfusion. These methods are non-invasive. The common finding is hyperperfusion during an acute stage and hypoperfusion in the chronic phase of SLE. Hyperperfusion may be caused by dilation of cerebral arteries and increased microvascular permeability in the lesion area. Hypoperfusion could be associated with cerebral cytotoxic edema, cortical atrophy and gliosis. Lesions mainly occur in the cerebral cortex and subcortical white matter regions with a predilection to the posterior brain areas, not limited to arterial territories and migratory [7,8,24]. In MRI examination, three stages of SLLs can be observed. The T1-weighted sequence shows hypointensity of lesions in the acute stage, hyperintensity in the subacute stage and decreased signal during the chronic phase. T2-weighted is the inverse of T1-WI. DWI signal is gradually amplified, which differentiates acute and subacute stages. MRS is a specific technique characterized by increased lactate peak in both stages, acute and subacute. PWI reveals hypoperfusion during a subacute phase [7,8,24].

## 4. Conclusions

SLEs are dominant phenotypic features of various mitochondrial disorders (MIDs). However, every case of SLEs needs to be distinguished from ischemic stroke. It has to be confirmed that SLLs and ischemic stroke changes may coexist. It is worth adding that, for example, MELAS patients may develop ischaemic stroke independent of an SLE due to atrial fibrillation, atherosclerosis, systolic dysfunction, arrhythmias, arterial hypertension, smoking, or low output failure. In SLE cases, we could confirm the SLLs using MRI. SLLs change over time after an episode and run through three stages: acute, subacute and chronic. In the acute stage, cerebral MRI shows hyperintensity on DWI. In the subacute stage, areas of cytotoxic edema enlarge and may be particularly found in the cortex. In the chronic stage, SLLs often display gyriform linear T1-hyperintensity consistent with laminar cortical necrosis [1,5,7,8,24].

There is no single criterion standard diagnostic test for mitochondrial disease. EEG changes are not specific in mitochondrial syndromes. Similar studies have been carried out to investigate EEG abnormalities occurring during an SLE. EEG shows periodic sharp waves in the left posterior region matching with the MRI lesion. Epilepsy during an SLE usually requires antiepileptic drug treatment, but SLE without epilepsy is hard to treat, and it probably does not react to any conventional therapy [16,22,25,26,29,30,31,32,33].

SLEs without seizures are treated with l-arginine, succinate, or citrulline. A supportive method is a ketogenic diet. The ketogenic diet is a high-fat, low-carbohydrate diet, so the main aim of its use is to stimulate fatty acid utilization by mitochondrial beta-oxidation, which produces ketone bodies that provide an alternative energy source for the brain and other tissues. Ketone bodies are metabolized to acetyl-CoA, which feeds into the Krebs cycle and then to the respiratory chain/mitochondrial oxidative phosphorylation system (OXPHOS) to generate ATP, and may at least partially bypass complex I. About 30% of childhood-onset MIDs are due to complex-I deficiency. Most childhood-onset MIDs are due to mutations in nDNA-located genes. While 75% of the adult-onset MIDs are due to mtDNA mutations [1,5,9,13,25,29,30,31,32].

Seizures associated with an SLE have been most frequently reported in patients with MELAS and only in a single patient with KSS. The occurrence of seizures and headaches or migraine simultaneously are not frequent, but migraine-like headaches during an SLE may go along with epileptiform discharges on EEG without clinically manifesting seizures. If SLEs are accompanied by seizures or in case of epileptiform discharges on EEG, antiepileptic drugs (AEDs) should be added. However, some AEDs are mitochondria-toxic, so they should be avoided if possible. These are valproic acid, carbamazepine, phenytoin and phenobarbital. A less mitochondria-toxic AED is pregabalin. In case AEDs are ineffective, a cocktail of vitamins, cofactors and antioxidants may be implemented [22,23,24,25,26,29,30,31,32,33,98].

However, SLEs are generally associated with inherited neurometabolic disorders, stroke mimicking symptoms could also occur in other conditions, including infectious diseases, migraine, glucose metabolism disorders (hypo/hyperglycemia), neurological impairments due to iron deficiency or inflammatory diseases [99,100,101,102,103].

Inherited metabolic disorders are connected with multiorgan dysfunction and affect various tissues. It results in abnormalities in basic laboratory tests, genetic alterations and neuroimaging. Another investigation tool is connected with histopathological changes observed in various inborn errors of metabolism. Morphological abnormalities such as intracellular or extracellular deposits, macro- and microvasculopathy or mitochondrial damage could explain the possible pathogenetic mechanisms (laboratory test abnormalities and histopathological changes in Table 1, Table 2, Table 3, Table 4 and Table 5) [104,105,106,107,108,109,110,111,112,113,114,115,116,117].

During the whole diagnostic process in patients with focal neurological symptoms, it is essential to take into consideration the possibility of SLE, especially in the pediatric population. The overall prevalence of inborn errors of metabolism is quite high, even if the frequency of individual disorders is very low. This fact indicates the alertness in investigating the probable causes of acute neurologic impairments. It is crucial to implement accurate diagnostic methods to detect and distinguish SLEs from other acute neurological events due to differences in further therapeutic management.

## Figures and Tables

**Table 1:** This table shows characteristic features of various neurometabolic disorders.

Neurometabolic Disorder	Genetic and Molecular Basis Pathomechanism	Clinical Manifestations Laboratory Tests Abnormalities	MRI	EEG	Histopathological Changes	Onset and Prevalence	Treatment
MELAS	# 540000 MELAS was first associated with a heteroplasmic point mutation, an A-to-G transition at position 3243 in the *MT-tRNA-L (UUR)* Decreased activity of Complex I nicotinamide adenine dinucleotide–coenzyme Q reductase and Complex IV cytochrome c oxidase	SLE, epileptic seizures, height deficiency, dementia, episodes of headaches, metabolic acidemia due to increased lactate level, diabetes mellitus, hearing loss and muscle weakness	Acute stage of sSLEs includes gyral swelling, gyriform cortical diffusion restriction, subcortical white matter T2 FLAIR hyperintensity with elevated ADC values and elevated parenchyma lactate in both acutely affected and non-affected brain regions on MRI spectroscopy Chronic stage shows as gyral infarction evolve into areas of encephalomalacia, volume loss and progressive multifocal cerebral atrophy. Symmetric basal ganglia calcifications could also occur Focal brain lesions are often localized in the occipital and parietal lobes, especially in the subcortical and cortical areas	Abnormalities do not occur in all cases of MELAS syndrome, but the most frequent is epileptiform activity Interictal epileptiform activity includes spikes, sharp waves and paroxysmal fast activity and their combinations with slow waves, such as spike-and-waves complexes (spike followed by a slow wave) and polyspike-and-wave complexes (multiple spikes followed by a slow wave)	Mitochondrial proliferation succinate dehydrogenase (SDH)-reactive blood vessels in endothelium and perivascular muscle	Affecting children and young adults; typically present between 2 and 40 years of age *Prevalence:* 1–9 / 1,000,000 Frequency of SLEs in patients with MELAS 60–80%	Treatment of SLEs with or without epilepsy and with or without paroxysmal Activity on EEG during SLE includes application of L-arginine, carnitine, Coenzyme-q10 Succinate or citrulline and ketogenic diet Results suggest that L-arginine supplementation may reduce risk of SLEs occurrence or the severity of present episodes
KSS	# 530000 Deletion/duplication analysis of mtDNA in all cases of KSS Heteroplasmic large deletions of the mtDNA Neuropathological changes due to respiratory chain dysfunction	Pigmentary retinopathy (progressive vision impairment due to rod-cone dystrophy). Progressive external ophthalmoplegia (PEO), including ptosis. Cardiac conduction abnormality, including heart block Development —KSS could induce premature death due to cardiac conduction defects; systemic involvement also includes chronic progressive external ophthalmoplegia with ptosis SLEs generally occur spontaneously or due to neuronal hyperexcitability epileptogenic discharges SLEs can be triggered by drugs such as phenytoin or zonisamide			Neuropathology reveals gliosis of the basal ganglia and neuronal degeneration Iron-containing pigment deposition in the caudate nucleus ad pallidum Spongy lesions in the cerebral white matter, brainstem and cerebellum		
			Brain MRI may show leukoencephalopathy, often associated with cerebral or cerebellar atrophy and/or basal ganglia and brainstem lesions KSS is characterized by distinctive neuroradiological abnormalities that occur in the cerebellum and brainstem but also in the diencephalon (thalamus), the striatum and the supratentorial white matter, especially subcortical T2-weighted vasogenic edema	Slow background rhythm		The onset is usually before 20 years of age Onset of SLEs: can occur in early childhood; average age is adolescence or adulthood, about 40 years of age *Prevalence:* 1–3 / 1,000,000	
MERRF syndrome	OMIM *#545000* mitochondrial inheritance Most cases have the common point mutation *m.8344A > G* in the MTTK gene Dysfunction of endothelium, angiopathy	Spasticity, myoclonic epilepsy, ataxia, generalized epilepsy, dementia loss of hearing, muscle weakness, exercise intolerance Ptosis, optic nerve atrophy, pigmentary retinopathy, optic neuropathy, cardiomyopathy, WPW syndrome, peripheral neuropathy Elevation of pyruvate or/and lactate serum level	Cerebral atrophy, cerebellar atrophy, high T2 white matter signal, globus pallidal atrophy and dentate nucleus calcification	Slowing of the background activity Generalized epileptiform discharges could be detected	Damage of mitochondria in skeletal muscle fibers and small blood vessels Deposits of calcium in vascular walls	Onset at childhood or adulthood Prevalence less than 1:100,000 Frequency of SLEs in patients with m8344A > G mutations 3–4% of patients	Symptomatic management, unavailability of specific treatment methods
LS	autosomal recessive inheritance mitochondrial mutations OMIM *#256000* Depletion of ATP energy, gliosis, excitotoxicity, reactive oxygen species (ROS) production. Increased lactate level leads to hypoxic encephalopathy and pathological change observed in patients with LS	Neurological manifestations such as hypotonia, ataxia, dystonia, nystagmus, strabismus, ophthalmoplegia, retinopathy, ptosis, epileptic seizures, retardation of development, dysfunction of the cardiorespiratory system dysfunction, gastrointestinal and hepatic impairments an increase in lactate blood and/or CSF levels	Bilateral focal necrotic lesions, mainly localized in the area of the thalamus, the brainstem and posterior parts of the spinal cord	EEG abnormalities - Focal seizures slower background activity with epileptic discharges	Vacuolization gliosis Vascular proliferation in brainstem and basal ganglia	Children at the age of 2 years or younger Prevalence: 1:36,000–40,000	No specific treatment Supplementation of thiamine or riboflavin
SSADH-D	4-hydroxybutyric aciduria, OMIM #271980 Monogenic autosomal recessive disorder of the γ-amino butyric acid (GABA)	Epileptic seizures development: develop epileptic seizures, ranging from absence seizures to generalized forms of epilepsy Elevated levels of GHB(gamma-hydroxybutyric acid) in urine, plasma and cerebrospinal fluid.	MRI— increased T2-weighted MRI signal affecting the globus pallidus, cerebellar dentate nucleus and subthalamic nucleus with variable cerebral/cerebellar atrophy	EEG abnormalities are generalized and focal epileptiform discharges, photosensitivity	Histology of the cortex and hippocampus revealed mild to moderate reactive astrogliosis.	Late childhood Very rare about 180 cases around the world	No curative therapy
DHPR	Autosomal recessive disorder of tetrahydrobiopterin (BH4) synthesis. OMIM#261630 mutations in the *QDPR* gene, leading to the deficiency of q-dihydropteridine reductase activity It causes an absolute reduction in tetrahydrobiopterin (BH4) levels and an impairment of phenylalanine to tyrosine conversion	Developed movement disorders and epilepsy Labolatory test shows hyperphenylalaninemia	Hyperintense areas in white matter on T2-weighted MRI Images in the regions of cystic lesions or diffused	Hypsarrhythmia paroxysmal activity	No DHPR activity detectable in peripheral blood cells, erythrocytes and leukocytes	Prevalence extremely rare	Treatment—low Phe diet, lifelong biogenic amine replacement therapy is given as L-Dopa and 5-hydroxytryptophan (5-HT)
CPS1 deficiency	OMIM *#237300* Autosomal recessive inheritance 2q34 *MIM 608307*	The lack of the CPSI enzyme results in hyperammonemia poor feeding, vomiting, somnolence, irritability ataxia, cerebral edema, seizures, mental retardation	Features of metabolic leukoencephalopathy abnormalities in subcortical white matter, caudate nuclei, dorsal part of the thalamus, hemisphere of the cerebellum Cortical injuries, including acute ischemia, ventricular dilatation and myelination defects	Increased background activity Abnormal spikes Diffused discharges	Measurement of CPSID1 enzyme activity on cells obtained from a liver biopsy can confirm the diagnosis	Mainly neonatal-onset	Low-protein diet hemodialysis L-arginine, L-carnitine, benzoate sodium supplementation
OTC deficiency	OMIM *#311250* X-linked inheritance Xp11.4 OTC *MIM 300461*	Hyperammonemic encephalopathy, seizures, vomiting Classic presentation in homozygous males in first weeks after birth	Extensive abnormality in signals of bilateral cerebral cortex, basal ganglia and thalamus Cerebral swelling	Multifocal ictal and interictal discharges	Biopsy shows decreased OTC enzyme activity in the liver	Typically occurs in the few first days of life May occur later in childhood or adulthood	Protein restriction hemodialysis to remove accumulated metabolites
Citrullinemia	OMIM *#215700* Autosomal recessive inheritance 9q34.11 ASS1 *MIM 603470*	Cirrhosis, hepatomegaly cerebral edema, ataxia, seizures, mental disability, developmental retardation, hyperammonemia, respiratory alkalosis	T2-weighted images—hyperintensity in the cerebral white matter, cingulate gyri, temporal areas, insula, pons Focal cerebral edema	EEG abnormalities include multifocal spikes, repeated paroxysmal activity	Liver histology reveals fatty change and fibrosis.	Childhood or adult onset Prevalence: 1:57 000	Dietary protein restriction sodium benzoate, sodium phenylacetate, arginine implementation

**Table 2:** This table shows characteristic features of various neurometabolic disorders—organic acidurias.

Organic Acidurias	Genetic and Molecular Basis Pathomechanism	Clinical Manifestations Laboratory Tests Abnormalities	MRI	EEG	Histopathological Changes	Onset and Prevalence	Treatment
Methylmalonic acidurias	Mutation: MMUT, MMAA, MMAB, MCEE, MMADHC Autosomal recessive, deficiency of the enzyme methylmalonyl-CoA mutase *MIM #251000* vascular dysfunction due to metabolic abnormalities mitochondrial impairments suggested inhibition of mitochondrial mechanisms by methylmalonate/metabolites	hypotonia seizures tremor developmental retardation vomiting hepatomegaly respiratory system dysfunction secondary increased level of ammonia ketonemia increased level of glycine acidemia aciduria	Bilateral pallidal lesions	slowed background activity	mitochondrial subsarcolemmal accumulation normal activities of respiratory chain enzymes brain atrophy decreased myelination hemorrhagic and necrotic lesions spongy alterations in the white matter, cortex of the cerebrum and cerebellum, brainstem, astrogliosis in the brainstem, hippocampus	1:50,000– 1:100,000	Hydroxycobalamin trial after stroke-like episode, infusion of glucose, carnitine supplementation, protein-restricted diet, amino-acid aid vitamins supplementation
Propionic aciduria	PCCA, PCCB OMIM #*606054* deficiency of propionyl CoA carboxylase activity affecting vascular endothelial function multiorgan pathology mitochondrial impairments	Encephalopathy, failure to thrive, chronic vomiting, focal neurologic signs, hemiparesis, hemiplegia, cardiomyopathy, hepatomegaly, renal dysfunction, vision problems metabolic acidosis hyperammonemia lactate, alanine and glutamine increased fluid/plasma ratios anemia, neutropenia, thrombocytopenia	initially normal and bilateral basal ganglia T2 hyperintensity with increased 18-Fluoro-2-deoxyglucose uptake in the basal ganglia and thalami and, finally, basal ganglia atrophy with decreased uptake	Comb-like rhythm in propionic aciduria encephalopathy, EEG during acute metabolic decompensation in PA may simply show severe, generalized diffuse slowing but returns to normal when metabolically stable	deposits of proteins in the liver, kidney, fatty tissue, skin degeneration observed in brain tissue	prevalence: 1:105,000–1:130,000	hydroxocobalamin trial, infusion of glucose, carnitine supplementation protein-restricted diet, amino acid and vitamins supplementation
Isovaleric aciduria	isovaleryl-CoA dehydrogenase deficiency OMIM *243500* depletion of energy due to inactivation of isovaleryl-CoA dehydrogenase	vomiting dehydration lethargy acidemia aciduria increased level of ammonia pancytopenia	T1-weighted MRI imaging shows hypointensity of bilateral globus pallidus T2-weighted and FLAIR MRI imaging shows hyperintensity of bilateral globus pallidus T1 hypointensity and T2 hyperintensity in the area of supratentorial white matter- neonatal acute stages of disease			neonatal or infantile/ later-onset prevalence: 1:250,000	

**Table 3:** This table shows characteristic features of Fabry’s disease and cystinosis.

Disorder	Genetic Basis and Molecular Basis Pathomechanism	Clinical Manifestations Laboratory Tests Abnormalities	MRI	EEG	Onset and Prevalence	Histopathological Abnormalities	Treatment
Fabry’s disease	X-linked lysosomal storage disorder subnormal activity of the alpha-galactosidase A, accumulation of glycolipids—globotriaosylceramide (Gb3) mainly in lysosomes *MIM# 301500* endothelial dysfunction, prothrombotic state higher production of ROS	growth retardation corneal and lenticular complications cardiovascular impairments renal failure obstructive respiratory disease nausea, vomiting, gastrointestinal complications anemia neuromuscular features TIA, seizures, SLE, stroke, autonomic failure anemia proteins in urine deficiency of alpha-galactosidase in plasma increased level of GB3 and lyso-GB3	dolichoectasia of the basilar artery bilateral T1-WI hyperintensity of the pulvinar white matter lesions are rare in patients under 50 years of age WML occurs in about 42.81% of patients with FD		typical occurrence before 10 years of age atypical late-onset adulthood prevalence: 1:20,000– 1:60,000	deposition of glycosphingolipids in the endothelium	intravenous enzyme replacement therapy chaperone therapy
Cystinosis	bi-allelic mutations in the CTNS gene 17p13.2-located autosomal recessive *MIM # 219800* accumulation and crystallization of cystine	hepatosplenomegaly renal failure myopathy cerebral atrophy acidosis aminoaciduria electrolytes level changes (hypokalemia, hyponatremia, hypophosphatemia) hematuria glucose and proteins in urine	cortical or central atrophy decreased gray matter volume in the left middle frontal gyrus white matter hyperintensity		occurs in the first year of life 1:100,000– 1:200,000	lysosomal accumulation of cystine	cysteamine bitartrate implementation

**Table 4:** This table shows characteristic features of TRMA.

Disorder	Genetic and Molecular Basis Pathomechanism	Clinical Manifestations Laboratory Tests	MRI	EEG	Histopathological Abnormalities	Onset and Prevalence	Treatment
TRMA	# 249270 autosomal recessive mutation in the *SLC19A2* gene respiratory chain dysfunction energy depletion reduction in oxidative phosphorylation, induction of oxidative stress secondary dysfunction of molecular activities in various tissues	non-autoimmune, early onset diabetes mellitus anemia sensorial deafness stroke and stroke-like episodes megaloblastic anemia dysfunction of glucose metabolism	reversible metabolic lesions	focal discharges	degeneration of peripheral nerves	prevalence: 30 families around the world about 100 cases have been reported	high doses of thiamine

**Table 5:** This table shows characteristic features of congenital disorders of glycosylation—PMM2 CDG.

Congenital Disorder of Glycosylation	Genetic Basis Pathomechanism	Clinical Manifestations Laboratory Tests Abnormalities	MRI	EEG	Histopathological Changes	Onset and Prevalence	Treatment
PMM2 CDG phosphomannomutase deficiency	* CACNA1A * mutations *MIM# 212065* (*601785OMIM) gain-of-function mutation in voltage-gated CaV2.1 channel glycosylation disorder leads to dysfunction in molecular cell activities such as protection against proteolysis or pathogens, enzymes, receptors activity	mono-/hemiparesis confusion seizures hyperpyrexia ataxia, developmental retardation, peripheral neuropathy, cerebellar abnormalities triggers of SLE: infectious diseases and head injury					
			cerebellar atrophy following extension of the 4th ventricle SLLs localized in occipital, parietal and temporal regions subcortical white matter white matter increased T2 signal in supra- and infratentorial	asymmetric slow activity of the background delta waves activity low voltage beta rhythms TIRDA temporal intermittent rhythmic delta waves activity theta rhythms hemispheric slowing of the background	macro- and microvascular changes liver steatosis inflammatory infiltration fibrosis	Onset: infancy, neonatal age SLEs in about 20–55% of patients with PMM2 CDG prevalence: 1:20 000	symptomatic management include antiepileptic medications hydration acetazolamide in treatment of cerebellar syndrome
